# Electrically-Conductive Polyketone Nanocomposites Based on Reduced Graphene Oxide

**DOI:** 10.3390/polym12040923

**Published:** 2020-04-16

**Authors:** Esteban Alejandro Araya-Hermosilla, Marco Carlotti, Francesco Picchioni, Virgilio Mattoli, Andrea Pucci

**Affiliations:** 1 Center for Micro-BioRobotics, Istituto Italiano di Tecnologia Viale Rinaldo Piaggio 34, 56025 Pontedera (PI), Italy; marco.carlotti@iit.it; 2 Department of Chemical Product Engineering, ENTEG, University of Groningen, Nijenborgh 4, 9747AG Groningen, The Netherlands; f.picchioni@rug.nl; 3 Dipartimento di Chimica e Chimica Industriale, Università di Pisa, Via Moruzzi 13, 56124 Pisa, Italy; 4 CISUP, Centro per l’Integrazione della Strumentazione dell’Università di Pisa, Lungarno Pacinotti 43, 56126 Pisa, Italy

**Keywords:** reduced graphene oxide, polyketone functionalization, electrically-conductive nanocomposites

## Abstract

In this work, we investigated the functionalization of polyketone 30 (PK30) with glycyl-glycine (Gly-Gly) via the Paal–Knorr reaction with the aim of homogenously dispersing two types of reduced graphene oxide (rGO, i.e., lrGO and hrGO, the former characterized by a lower degree of reduction in comparison to the latter) by non-covalent interactions. The functional PK30-Gly-Gly polymer was effective in preparing composites with homogeneously distributed rGO characterized by an effective percolation threshold at 5 wt. %. All the composites showed a typical semiconductive behavior and stable electrical response after several heating/cooling cycles from 30 to 115 °C. Composites made by hrGO displayed the same resistive behaviour even if flanked by a considerable improvement on conductivity, in agreement with the more reduced rGO content. Interestingly, no permanent percolative network was shown by the composite with 4 wt. % of lrGO at temperatures higher than 45 °C. This material can be used as an ON–OFF temperature sensor and could find interesting applications as sensing material in soft robotics applications.

## 1. Introduction

Graphene has called high attention thanks to its excellent mechanical properties, thermal conductivity, and electronic transport properties [1,2,3,4]. However, the lack of high quality samples, elevated cost of production, poor dispersibility in solvents, and irreversible aggregation in several media have limited its use [5]. Instead, graphene oxide (GO) has replaced graphene in many applications due to low cost of production and dispersibility in water and polar organic solvents [6,7]. As graphene, it possesses a 2D structure but some of the carbons atoms lost the sp^2^ character being involved in the covalent linkage with hydroxyl, epoxide, and carbonyl groups generated during the oxidation of the graphene layer [8,9,10]. These differences are enough to affect the conjugation properties of the former and thus GO behaves as an insulator [11,12]. The electric properties of GO can be recovered by reduction treatments (producing reduced graphene oxide (rGO)), which partially restores the graphitic network of sp^2^ carbons [13,14]. Notably, the residual functional groups remained in the rGO structure make its dispersion easier in organic solvents [15] and increase the number of effective interactions also with several polymer matrices thus helping their homogeneous distribution in the solid matrix [16,17]. One can therefore take advantage of these features for the preparation of functional polymer nanocomposites [18,19,20] where rGO can provide enhanced electrical and mechanical properties [21,22,23,24,25]. In this sense, these materials have found a variety of applications such as supercapacitor electrodes [26], chemical sensor [27], and antibacterial scaffolds [28]. However, despite the better results when compared to graphene, obtaining good dispersions of rGO into the polymer matrices is still challenging: the absence of highly interacting functional groups in several polymers causes rGO aggregation, which limits the ultimate application of the derived composites [29]. 

**Figure 1:**
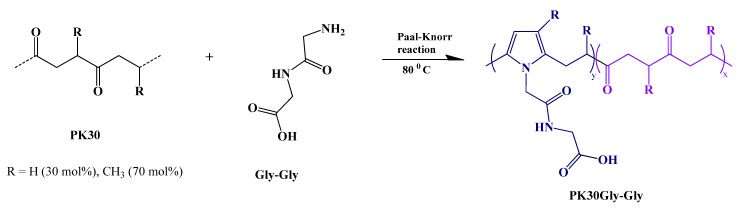
Scheme of Paal–Knorr reaction held by polyketone 30 (PK30) and glycyl-glycine (Gly-Gly).

Conversely, functional polymers able to provide strong and reliable non-covalent interactions with rGO, can result in composites characterized by enhanced mechanical, electrical, and thermal properties even at low filler content [30]. Our group has recently reported the high exfoliation ability of polyketone functionalized with aromatic [31] and hydroxyl pendant groups [32] towards multiwalled carbon nanotubes (MWCNTs): the resulting nanocomposites showed an effective percolation network at low MWCNTs concentration and high stable electrical response after several heating/cooling cycles, making them promising materials for temperature sensors. Based on these findings, we decided to investigate the dispersion of rGO in a polyketone functionalized by the Paal–Knorr reaction (Figure 1). Polyketones are a promising starting polymer for the synthesis of functional polymers through the chemical modification approach due to the presence of highly reactive 1,4-di-carbonyl moieties that react with primary amines yielding water-resistant *N*-substituted pyrrole units [33]. The Paal–Knorr is a versatile reaction as it can be carried out in bulk, without catalysts, with high yields and with water being the only by-product [34]. In addition, it may be also performed without solvent or in various organic solvents depending on the physical and chemical properties of the amine compound. It is tolerant to a number of primary amines making this easy synthesis a fast, cheap, and appealing approach to create polymers with almost any desired pendant functional group [31,32,35,36]. The resulting functional polymers find use in different applications such as self-healing materials [37,38], emulsions with adhesive properties [39,40], and coating materials [41].

The present work focuses on the synthesis of a functional polymer by chemical modification of alternating aliphatic polyketone with glycylglycine (Gly-Gly) via the Paal–Knorr reaction. The amide and carboxylic groups of Gly-Gly, along with the formation of the pyrrole groups, enable the polymer matrix to interact with rGO via non-covalent interactions (e.g., H-bonding and π–π interactions), thus promoting the formation of the nanocomposite without any chemical conversion of the functional moieties [42,43]. We, therefore, prepared a series of nanocomposites comprising different amount and types of rGO and characterized their final morphology and electric properties also in terms of the resistive behavior as a function of temperature. 

## 2. Experimental

### 2.1. Materials and Methods

Aliphatic polyketones composed by ethylene, propylene, and CO were synthesized according to a reported procedure [44,45] yielding a polyketone with the aliphatic part comprised of 30 mol% ethylene and 70 mol% propylene (PK30, Mw 4670 Da). Glycyl-glycine (Gly-Gly) (Sigma Aldrich, Milan, Italy, 97%) and 2,5-hexanedione (Sigma Aldrich, Milan, Italy, 98%) were used as received. Different reduced graphene oxide (rGO), i.e., lrGO and hrGO, respectively, were kindly provided by Abalonyx (Oslo, Norway). Briefly, rGO was prepared by introducing the dry graphene oxide powder into a quartz tube tubular oven (about 1 g/min). The graphene oxide flashes in the hot zone and is then transported out of the tube by means of a continuous flow of air and collected on a filter. Residence time in the hot zone is estimated to be about 2 s. The oven temperature was set at 430 and 625 °C, for lrGO and hrGO, respectively. Elemental analysis: lrGO = C 68.84%, H 1.41%, O 29.14%, and N 0.48%; hrGO = C 74.64%, H 1.09%, O 23.12%, and N 0.64%.

2,5-hexanedione was prepared as a model compound and aimed at the correct assignment of ^1^H-NMR signals after the Paal–Knorr synthetic process with Gly-Gly. The reaction between stoichiometric amounts of Gly-Gly and 2,5-hexadione was carried out in a 100 mL round-bottomed flasks equipped with a magnetic stirrer, a reflux condenser and a Heat-On™ Block System (Radleys Shire Hill, Saffron Walden, Essex, United Kingdom). First, 1.45 g of 2,5-hexadione (0.013 mol) and 1.72 g of Gly-Gly (0.013 mol) were placed in the flask. The reaction was carried out in ethanol at 80 °C under stirring (700 rpm) for 48 h in order to ensure reaction completeness. The solvent was removed at reduced pressure and the recovered material introduced in an oven at 40 °C for 48 h for complete dryness. ^1^H-NMR (400 MHz δ, CDCl_3_) = 2.2 ppm (s, 6H), 4.0 ppm (d, 2H, J = 5.8), 4.5 ppm (s, 2H), 5.8 ppm (t, 1H, J = 5.7 Hz), 5.87 ppm (s, 1H) [46]. 

### 2.2. Polyketone Modification

The functionalization of PK30 with Gly-Gly was carried out to reach 30% of conversion of polyketone di-carbonyl groups (Table 1). First, 47.192 g of PK30 were dissolved in 75 mL of ethanol in a 250 mL round-bottomed flasks equipped with a magnetic stirrer, a reflux condenser and a Heat-On™ Block System. Afterward, 14.21 g of Gly-Gly were added to the polymer solution. The reaction was carried out at reflux for 36 h. The polymer was filtered to collect the unreactive amine, re-dissolved in ethanol and filtered again and the procedure repeated three times. The solvent was removed at reduced pressure, and then placed in an oven at 40 °C for 48 h for complete dryness. The carbonyl conversion (*C_co_*), i.e., the molar fraction of 1,4-dicarbonyl units reacted via the Paal–Knorr reaction, was calculated on the basis of elemental analysis as the following:

(1) $$C_{co}=\frac{y}{y+x}\times 100\%$$

where *x* and *y* are the di-ketone and pyrrolic moles after conversion, respectively. *y* was determined as follows:

(2) $$y=\frac{wt\left(N\right)}{A_{m}\left(N\right)}$$

where *wt*(*N*) are the grams of *N* of the product as determined by elemental analysis and *A_m_*(*N*) is the atomic mass of N. *x* was then determined as follows:

(3) $$x=\frac{g_{prod}-y\times M_{w}^{y}}{M_{w}^{pk}}$$

where *g_prod_* is the gram of the product, $M_{w}^{y}$ the molecular weight of the pyrrolic unit and $M_{w}^{pk}$ the molecular weight of a 1,4 di-ketone unit. From the ratio between *C_co_* and the corresponding amount in alimentation ($C_{co}^{feed}$), the conversion efficiency η con be calculated as the following:

(4) $$\eta =\frac{C_{co}}{C_{co}^{feed}}\times 100\%$$

where $C_{co}^{feed}$ corresponds to:

(5) $$C_{co}^{feed}=\frac{Mol_{amine}}{Mol_{d-CO}}\times 100\%$$

with $Mol_{amine}$ are the moles of amine and $Mol_{d-CO}$ the moles of di-carbonyl units in alimentation.

### 2.3. Preparation of rGO/PK30-Gly-Gly Composites

The rGO/PK30-Gly-Gly composites were prepared by mixing PK30-Gly-Gly and lrGO or hrGO at different weight concentration (wt. %). First, PK30-Gly-Gly was dissolved in a round bottom flask containing 20 mL of chloroform under vigorous stirring at 50 °C for 20 min. The corresponding 4–8 wt. % for lrGO or 5–6 wt. % for hrGO amounts of rGO with respect to PK30-Gly-Gly was added to 20 mL of chloroform and tip sonicated for 5 min in an ice bath to avoid the loss of solvent. Afterward, the dispersion was added to the polymer solution and the system vigorously stirred at 50 °C for 24 h. After cooling to room temperature, the solvent was removed and the recovered rGO/PK30-Gly-Gly composite dried completely at 40 °C for 24 h.

### 2.4. Characterization

The elemental composition of rGO and polymers was determined by using an Elementar Vario Micro Cube for nitrogen, carbon and hydrogen.

^1^H-NMR spectra were recorded at room temperature in CDCl_3_ solution with a Bruker Avance DRX 400 spectrometer (Bruker, Billerica, MA, USA), using the residual solvent peak as internal reference.

ATR–FT–IR spectra were recorded by means of a Perkin-Elmer Spectrum One (San Francisco, CA, USA), within the 4000–650 cm^−1^ and averaged over 32 scans. 

Differential scanning calorimetry (DSC, TA Instruments, New Castle, DE, USA) was carried out by means of a TA DSC250 under N_2_ in agreement with previous studies [32,35]. 

Gel Permeation Chromatography (GPC) measurements were performed with an HP1100 Hewlett-Packard (Hewlett-Packard, Wilmington, Philadelphia, PA, USA) and in agreement with procedures previously reported [32,47].

Scanning transmission electron microscopy (STEM, Thermo Fisher Scientific, Hillsboro, Oregon, USA) was performed on rGO samples using a FEI Quanta 450 ESEM equipped with a field emission gun. Particle analysis was performed using the public domain ImageJ 1.52k software (National Institutes of Health, Bethesda, MD, USA).

Thermal degradation of rGO was analyzed via thermogravimetric analysis (TGA) with a Mettler Toledo TGA/SDTA851 instrument (Mettler Toledo, Columbus, OH, USA) under nitrogen flux (80 mL/min). All samples were tested in agreement with procedures previously reported [48,49].

Raman spectroscopy has been performed using a Horiba Jobin Yvon Xplora ONE confocal Raman microscope (Horiba Scientific, Horiba Italy, Roma, Italy). The wavelength of the excitation laser was 542 nm and the power of the laser was kept below 1 mW without noticeable sample heating. 

Microscopic morphology of the composite samples was observed by Scanning Electron Microscopy, by using a Dual Beam FIB/SEM Helios Nano-Lab 600i (Thermo Fisher Scientific, Hillsboro, OR, USA), 10 kV accelerating voltage and variable magnification. For SEM imaging, the samples were prepared by Au deposition (layer about 40nm) using AC sputtering.

The electrical measurements of the lrGo and hrGO solid dispersions were carried out according to literature reports [50,51].

The temperature dependent resistivity measurements were performed on square 6 mm × 6 mm samples with 1.05 mm of thickness, connected with two copper electrodes to the opposite edge of the square: the temperature control was obtained by placing the sample on a thin ceramic plate substrate (0.3 mm) equipped with a gold resistance (on the bottom side, not in direct contact with sample) connected to a controllable power supply module. The temperature was monitored with a k-thermocouple brought in contact with the sample; the resistance was continuously measured using a High precision multimeter (Model 187, Fluke, Fluke Corporation, Everett, WA, USA) connected with the copper electrodes. Measurements were performed modulating the temperature by changing the current applied to the ceramic heater and waiting for the temperature stabilization.

## 3. Result and Discussion

### 3.1. Polyketone Functionalized with Gly-Gly Groups via the Paal–Knorr Reaction

We prepared PK30-Gly-Gly by chemical modification of polyketone 30 with Gly-Gly via the Paal–Knorr reaction. The functionalization of polyketone yielded a *C_co_* of 29% as established from elemental analysis data (Table 1). The total di-carbonyl efficiency can be calculated by elemental analysis (*x* + *y*) using the relative content of nitrogen in the products. Moreover, PK30 shows a weight average molecular weight of 4670 with a dispersity index of 1.7, that are supposed to be mostly unchanged the functionalization process in agreement with the recent literature [47]. Nevertheless, attempts at measuring the molecular weight of the functionalized PK30 were not successful due to significant aggregation phenomena among the macromolecules during chromatography. 

**Figure 2:**
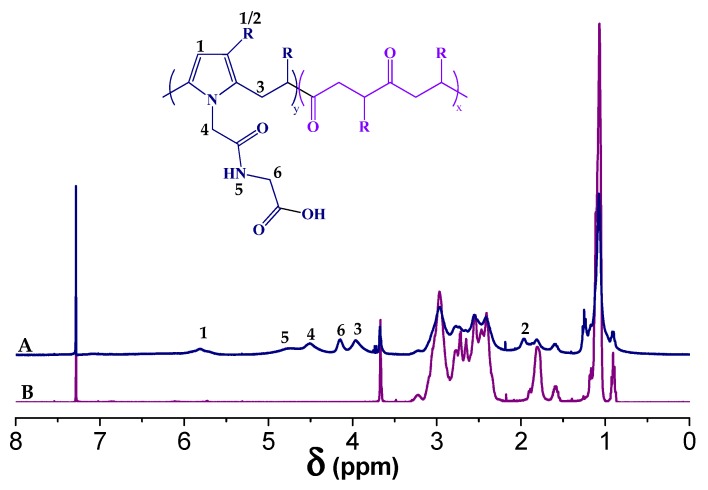
^1^H-NMR spectra of (**A**) PK30 functionalized with Gly-Gly and (**B**) pristine PK30 in CDCl_3_.

The PK30 functionalization with Gly-Gly is confirmed by ATR–FT–IR and ^1^H-NMR spectroscopies. Figure 2 shows the ^1^H-NMR spectra of PK30 before (B) and after (A) functionalization. The signals of the pyrrole rings closely correspond to those of the model compound that we described in the experimental section (Section 2.1). The success of the grafting process is indicated by the proton signals at 5.7 (H1), 2.0 (H2), and 3.97 (H3) ppm that we attributed to the pyrrole ring formed during the Paal–Knorr reaction, the methyl group, and methylene groups adjacent to it, respectively [34]. The methylene proton of the functional group was assigned at 4.15 (H6) and 4.51 ppm (H4), and the proton of the amide group at 4.75 ppm (H5), whereas the remaining signals between 2.3 and 2.9 ppm were attributed to the unreacted PK aliphatic part comprised of 30 mol% ethylene and 70 mol% propylene. 

**Figure 3:**
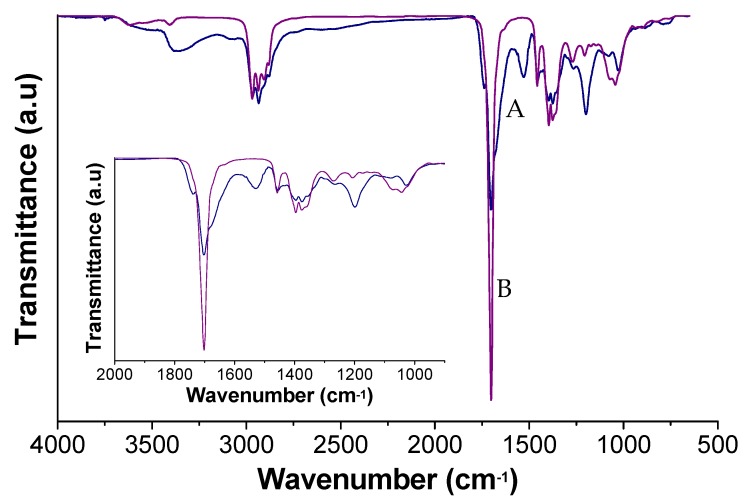
FT-IR spectra of (**A**) PK30 functionalized with Gly-Gly and (**B**) pristine PK30.

Figure 3 shows the ATR–FTIR spectrum of PK30 and PK30-Gly-Gly. After the Paal–Knorr reaction has occurred, the intensity of the carbonyl group signal (1700 cm^−1^) decreases, due to the disappearance of the 1,4-dicarbonyl moieties. In addition, two peaks appeared for PK30-Gly-Gly very close to 1700 cm^−1^ that correspond to the carbonyl groups of the carboxylic acid at 1745 cm^−1^ and the amide moiety at 1670 cm^−1^. The appearance of these two peaks, together with the decrease in intensity of the signal associated with the PK carbonyl indicates the successful modification of the starting polymer. We also assigned the wide weak peak from 3650 to 2000 cm^−1^ in PK30-Gly-Gly to the hydrogen bonding of the carboxylic groups. Moreover, the peak associated to the pyrrole units was found at 3100 cm^−1^ (C=C–H) whereas the N–H and C–N stretching of the secondary amide can be found at 3397 and 1209 cm^−1^, respectively. The weak signal at 1540 cm^−1^ may correspond either to stretching of C=N and C=C bonds or to both. Finally, stretching bands of aliphatic C–H of PK backbone and functional groups appeared between 2969 and 2873 cm^−1^ [46].

Another evidence that the chemical functionalization was successful can be found in the higher glass transition temperature (*T*_g_) we measured for PK30-Gly-Gly (31.6 °C, Figure S1) compared to its precursor PK30 (−17.7 °C). This is possibly due to a combination of effect: a) the formation of pyrrole units after the Paal–Knorr reaction and the presence of hydrogen bonds between the Gly-Gly groups of different polymer chains [35,37], both contributing in the rigidity enhancement of the polymer matrix.

### 3.2. Preparation and Characterization of rGO/PK30-Gly-Gly Composites

We used PK30-Gly-Gly as supporting polymer matrix for two types of rGO (i.e., lrGO and hrGO) that were obtained from GO by means of thermally reduced processes at different temperatures, i.e., 430 and 625 °C, for lrGO and hrGO, respectively. PK30-Gly-Gly (Figure 1) was specifically designed as to show the presence of pyrrole rings along the backbone and the amide + carboxylic acid as pendant groups thus possibly allowing a strong and effective (H-bonding) interactions with graphitic fillers [32,52]. In particular, lrGO was characterized by a lower degree of reduction in comparison to hrGO, according to the different reduction temperature. This was confirmed by the different carbon content as determined by elemental analysis. Notably, on passing from lrGo to hrGO, the amount of carbon increased from 68.84% to 74.64%. Moreover, thermogravimetric analysis (TGA, Figure S2) evidenced a different degradation behaviour of the two types of rGO as well as a distinct residual mass. The weight loss is directly correlated to the amount of residual oxygen-containing groups, thus rGO with a larger reduction extent should display a lower weight loss. Indeed, lrGO had a residual mass of 68.9% in comparison with the 74.9% of hrGO, in strict agreement with the elemental analysis. Moreover, lrGO showed an inflection point at 218 °C that corresponds to the thermal decomposition of the oxygen-containing residual groups [53], which resulted much less pronounced in the hrGO sample. The surface size of the flakes for the two rGOs was evaluated by STEM (Figure S3) and found to be 2.95 ± 0.35 and 2.52 ± 0.74 μm^2^ for lrGO and hrGO, respectively. Raman spectroscopy is a versatile technique used for the structural characterization of graphitic materials including graphene, graphene oxide, and reduced graphene oxide. It is well reported that the most prominent features in a Raman spectrum of rGO are the G and D bands (Figures S4 and S5). The G-band at about 1580 cm^−1^ is an intrinsic feature of graphene and related to the planar vibration of carbon atoms in most sp^2^ graphitic materials. Conversely, the disorder-induced D-band at about 1340 cm^−1^ is attributed to the scattering from defects breaking the basic symmetry of the graphene sheet [54,55]. The large contribution at about 2900 cm^−1^, is possibly attributed to the combination of the first overtone of the D band (2D band) and D + G band. Notably, the ratio of D and G bands peak intensities (I_D_/I_G_) is a common index for the extent of defects on different qualities of rGO. On passing from lrGO (Figure S4) to hrGO (Figure S5) it was found that the I_D_/I_G_ ratios of rGO samples changed from 1.09 to 1.20 even if the contribution of the 2D + G + D bands did not substantially change. This result was attributed to the combination of two opposite phenomena: the first is the increased reduction extent on passing from lrGO to hrGO that favors the restoration of a larger amount of ordered graphitic structure, thus enhancing the I_D_/I_G_ ratio. The second is the loss of carbon content that possibly occurs for thermally-activated reduction processes at higher temperature that, in turn, adversely affects the graphitic layer extent [56]. This last hypothesis is in agreement with the average surface size determined by STEM experiments. 

The different reduction extent between lrGO and hrGO was also reflected on their electrical conductivity. Aliquots (100 μL) of rGO chloroform dispersions were drop-cast onto gold plated electrodes supported on a Kapton^®^ film and the electrical resistance was measured after the complete evaporation of the solvent. Electrical resistances, determined as the average of three distinct depositions, of 81.7 ± 15.41 MΩ and 38.11 ± 2.11 MΩ were measured for lrGO and hrGO, respectively, thus confirming that the larger reduction degree is associated to a higher graphitic conductive extent.

**Figure 4:**
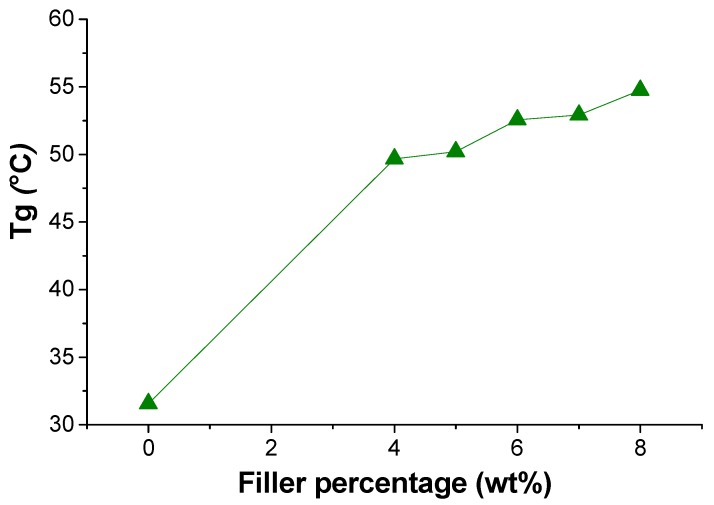
Glass transition temperature (differential scanning calorimetry (DSC)) of pristine PK30-Gly-Gly and of the respective composites containing lower degree of reduction graphene oxide (lrGO) as the filler.

rGO/PK30-Gly-Gly composites were then prepared by solvent-aided mixing of the polymer with different amounts of rGO and characterized by means of various techniques. DSC experiments of PK30-Gly-Gly composites comprising lrGO or hrGO showed that they have a higher *T*_g_ in comparison to PK30-Gly-Gly alone (Figure S1 shows the experimental curves used to derive the data). Figure 4 shows the *T*_g_ increase for the lrGO/PK30-Gly-Gly composites as a function of the filler concentration. The rGO/PK30-Gly-Gly composites with hrGO displayed a similar trend with *T*_g_ values of 52.7 °C and 53.5 °C for the 5 and 6 wt. % content, respectively. According to the literature, the increase of the *T*_g_ in nanocomposite is tightly related to the enhanced system viscosity due to the interfacial interaction between the polymeric matrix and the filler [57,58]: in our case the pendant functional groups and the pyrrole rings of the polymer both effectively interact with the rGO, thus limiting the polymer mobility [59,60,61]. Overall, these results prove the good affinity between the filler and the polymer. 

**Figure 5:**
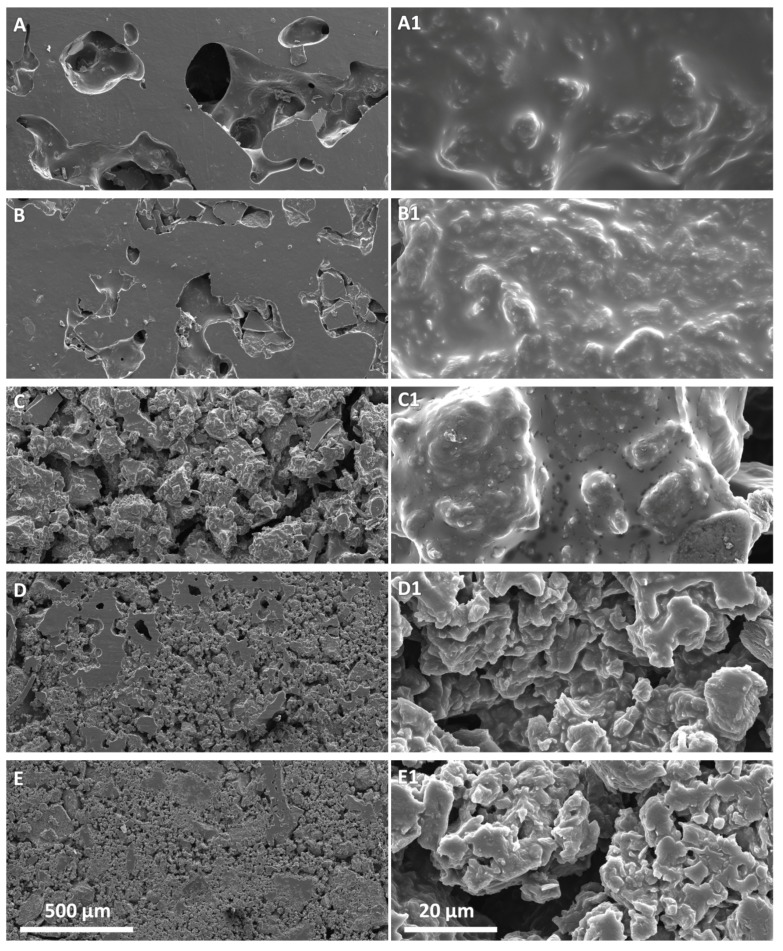
Scanning Electron Microscopy (SEM) micrographs at different magnification of the nanocomposite composed by PK30-Gly-Gly and lrGO at different weight percentage. (**A**,**A1**) 4 wt. %, (**B**,**B1**) 5 wt. %, (**C**,**C1**) 6 wt. %, (**D**,**D1**) 7 wt. %, and (**E**,**E1**) 8 wt. %. Left pictures scale bar 500 μm, right pictures scale bar 20 μm.

**Figure 6:**
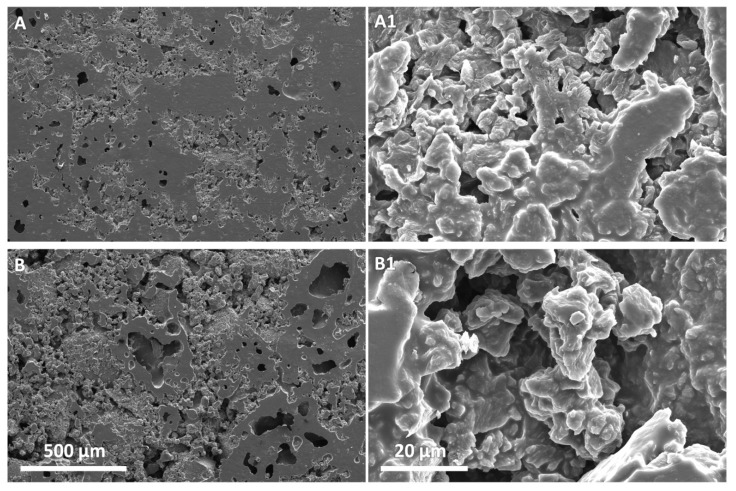
SEM micrographs at different magnification of the nanocomposite composed by PK30-Gly-Gly and hrGO at different weight percentage. (**A**,**A1**) 5 wt. % and (**B**,**B1**) 6 wt. %. Left pictures scale bar 500 μm, right pictures scale bar 20 μm.

We also evaluated the morphology of the composite material and the dispersion degree of rGO by SEM microscopy (Figure 5). Pictures in Figure 5 show a constant morphological change of the material as the amount of lrGO increase. The composite with less content of lrGO (4 wt. %) displays a smoother surface as also revealed at higher magnifications (Figure 5A,A1). As soon as the lrGO content increases a more porous structure is evidenced, especially at the highest 7 and 8 wt. % (Figure 5C,D). Nevertheless, the SEM micrographs do not evidence the presence of a significant phase separation between the composite components and lrGO appears as homogeneously distributed graphitic filler within the PK30-Gly-Gly matrix. More than that, at the highest magnification lrGO appears homogeneously distributed within the interacting PK30-Gly-Gly thus possibly suggesting the formation of effective percolation pathways (Figure S6). The composites comprising 5 and 6 wt. % of hrGO show similar morphology (Figure 6) and appear similar to those prepared with the same concentration of lrGO as well (Figure 5B,C and Figure S7). 

**Figure 7:**
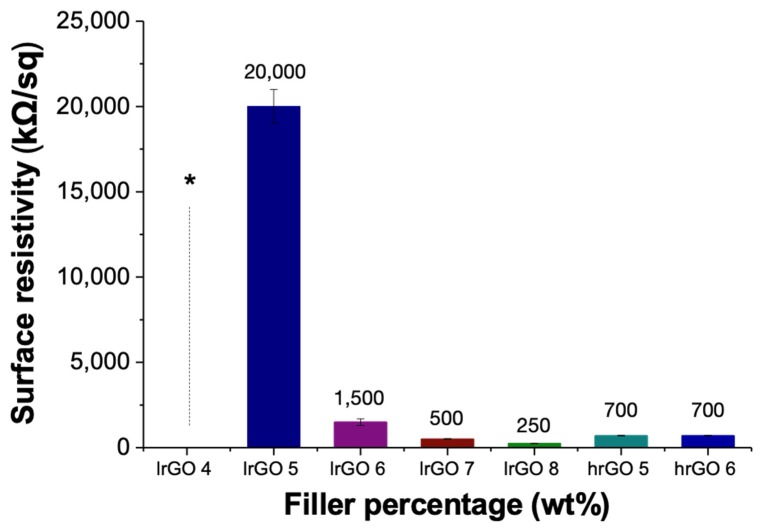
Surface resistivity of the nanocomposites composed by PK30-Gly-Gly and lrGO or higher degree of reduction graphene oxide (hrGO) at different weight percentage at 30 °C. * ≥ 500 MΩ/sq. Sample thickness of 1.05 mm.

Figure 7 summarizes the resistivity of the samples composed by PK30-Gly-Gly and lrGO. As expected, the resistance decreased when increasing the rGO content dispersed into the polymer. While the composite with 4 wt. % of lrGO displayed a surface resistivity higher than 500 MΩ/sq at 30 °C, an effective percolation pathway was reached with 5 wt. % of lrGO. Moreover, significant decrease in terms of surface resistivity occurred on going from 5 to 8 wt. % of the graphitic filler up to values of 250 kΩ/sq. 

To evaluate the effect of different degrees of reduction in the rGO filler, we tested samples containing 5 and 6 wt. % of hrGO with a similar procedure. The results are summarized in the Figure 7. Unlike the previous case, the increment in rGO from 5 to 6 wt. % did not produce any evident variation in surface resistivity that was of 500 kΩ/sq for both composites. Nevertheless, these values appeared remarkably lower that those measured from the corresponding lrGO/PK30-Gly-Gly composites, in agreement with the more reduced rGO content in the hrGO sample.

### 3.3. Composite Resistance Sensitivity to Temperature

**Figure 8:**
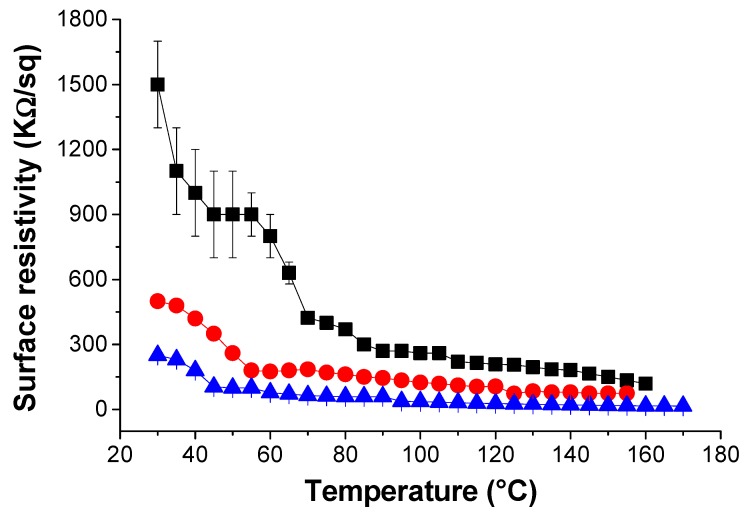
Surface resistivity of the nanocomposite composed by PK30-Gly-Gly and lrGO at different temperatures and filler concentration. (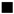) 6 wt. %. (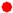) 7 wt. %. (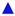) 8 wt. %. Sample thickness of 1.05 mm.

As a semiconductor, the conductivity of rGO increases upon increasing the temperature. Figure 8 shows the resistivity vs. temperature plot for the different lrGO/PK30-Gly-Gly composites (6, 7, and 8 wt. %). For each sample, the resistance lowered upon heating. In addition, the composites displayed a marked reduction in resistivity after 50 °C, which one can relate to the *T*_g_ of the same composites (Figure 4). Also, the composite with 4 wt. % started to show resistivity values from 45 °C (Figure S8). This indicates that above the *T*_g,_ the polymeric matrix allows for an increased mobility of the rGO flakes, thus reaching a temporal network where the electrons can flow through the material [62].

**Figure 9:**
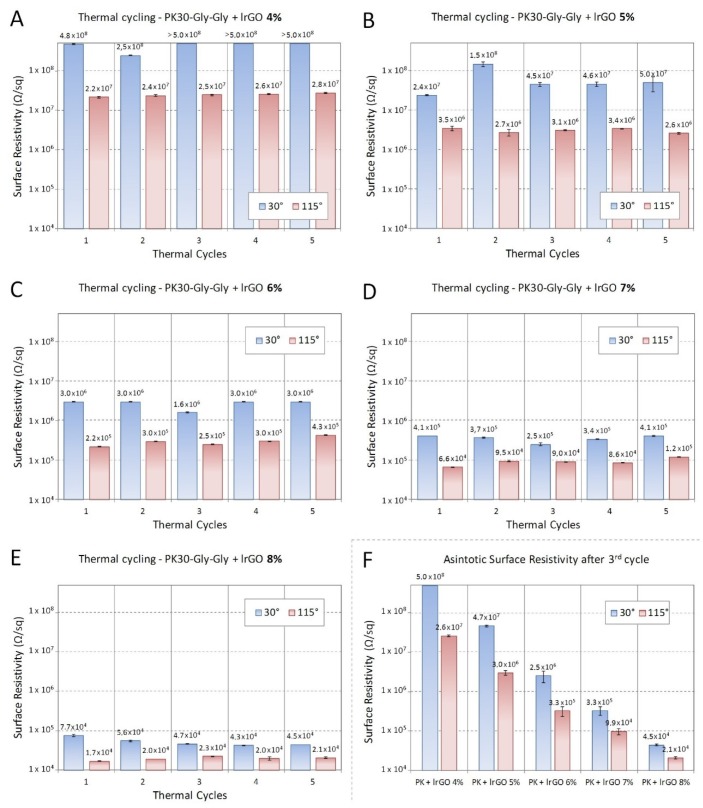
Surface resistivity measured at 30 °C (light blue) and 115 °C (light red) upon repeated cycles of the nanocomposite composed by PK30-Gly-Gly and lrGO. (**A**) 4 wt. %, (**B**) 5 wt. %, (**C**) 6 wt. %, (**D**) 7 wt. %, and (**E**) 8 wt. %. Sample thickness of 1.05 mm. (**F**) Asymptotic surface resistivity as average over last three thermal cycles.

An important characteristic to evaluate is the material fatigue upon heating and cooling cycles. In Figure 9, we show the resistivity variation of the lrGO/PK30-Gly-Gly composite during five heating and cooling cycles from 30 to 115 °C. In general, we observed that the resistivity of the materials was reliably restored across every cycle. Indeed, the composites with lrGO at concentrations of 4 wt. % returns to be an electrical insulator as the heating system is switch off. This evidence suggests that these composites could be used as temperature sensors over the investigated operating range, more preferably between room temperature and 80 °C, i.e., where the surface resistance variation was maximum. 

**Figure 10:**
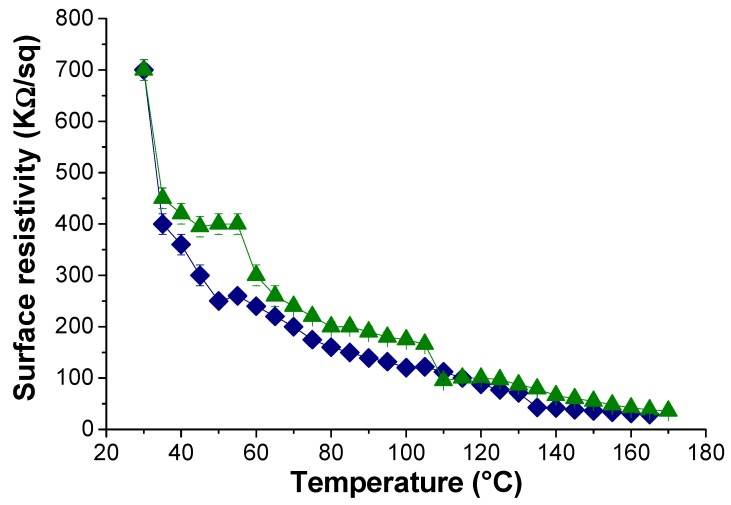
Resistivity of the nanocomposite composed by PK30-Gly-Gly and hrGO at different temperatures. 5 (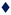) and (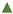) 6 wt. % of filler concentration.

**Figure 11:**
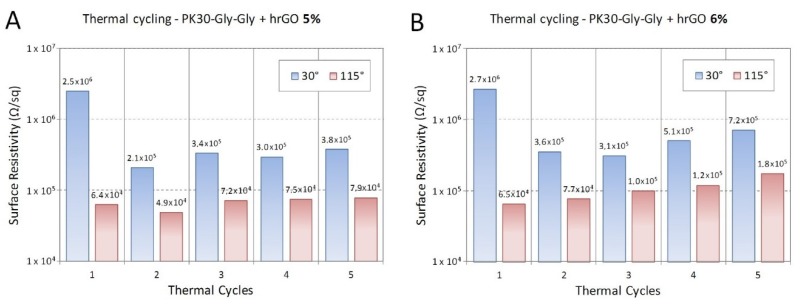
Surface resistivity measured at 30 °C (light blue) and 115 °C (light red) upon repeated cycles of the nanocomposite composed by PK30-Gly-Gly and hrGO. 5 (**A**) and (**B**) 6 wt. % of filler. Sample thickness of 1.05 mm.

The same experiment was repeated for the hrGO/PK30-Gly-Gly composites (Figure 10 and Figure 11). Despite the variation of the filler, these composites possess the same behaviour of the ones comprising lrGO.

## 4. Conclusions

We have shown the straightforward preparation of polymeric nanocomposites comprising rGO using the novel functionalized PK30-Gly-Gly polyketone bearing pyrrole rings in the backbone and amide and carboxylic acid as pendant groups. We synthesized the polymer by the chemical modification of an alternating aliphatic polyketone via the Paal–Knorr reaction. The former was able to interact with rGO by effective non-covalent interactions, thus facilitating the exfoliation process without damaging the one-dimensional arrangement of rGO. We also easily modulated the electrical resistance of the composite by increasing the concentration of rGO in the mixing process. Indeed, resistivity measurements supported by SEM investigations demonstrated that the effective percolation pathway is achieved from an rGO concentration of 5 wt. %. Heating and cooling cycles showed that the conductive network is preserved, suggesting a high stability of the rGO dispersion within the polymeric matrix. Interestingly, we found that the composite with 4 wt. % lrGO (below the percolation threshold) can act as an ON–OFF system with temperature, allowing a low resistance only when the temperature is higher than the *T*_g_ of the composite and otherwise behaving as an insulator. Overall, these results support the use of functionalized polyketones as the matrix for rGO nanocomposites. Such materials may have a predominant role in the development of the next generation of soft robotics devices, in which one can modulate mechanical and electrical properties by using diverse inputs such as temperature and deformation. 

## Figures and Tables

**Table 1:** Elemental analysis of pristine PK30 and PK30 after functionalization with Gly-Gly.

Sample	*x*(%)	*y*(%)	*C_co_*(%) ^1^
PK30	100	-	-
PK30_x_Gly-Gly_y_	70.8	29.2	29.2

^1^ Di-carbonyl conversion (CO %) obtained from EA.

## Supplementary Materials

The following are available online at https://www.mdpi.com/2073-4360/12/4/923/s1, Figure S1: DSC first cycle after thermal history erases of PK30Gly-Gly29 and its respective composite with lrGO and hrGO. The first curves are not shown since they are carried out to remove the thermal history of the polymer and composites; Figure S2: TGA analysis of a) lrGO and b) hrGO; Figure S3: STEM micrograph of a) lrGO and b) hrGO; Figure S4: Raman spectrum of lrGO; Figure S5: Raman spectrum of hrGO; Figure S6: SEM picture of the PK30-Gly-Gly nanocomposite containing 7 wt% lrGO; Figure S7: SEM picture of the PK30-Gly-Gly nanocomposite containing 6 wt% of hrGO; Figure S8: Surface resistivity of the nanocomposite composed by PK30-Gly-Gly and lrGO at different temperatures and filler concentration. (A) 4 wt%. (B) 5 wt%. Sample thickness of 1.05 mm.
