# Supplementary material for: Electrically-Conductive Polyketone Nanocomposites Based on Reduced Graphene Oxide
Source: Polymers (Basel). 2020 Apr 16;12(4):923. doi: 10.3390/polym12040923 (PMC7240681; doi:10.3390/polym12040923)
Supplement: Supplementary file 1 [file polymers-12-00923-s001.pdf]

polymers

Supplementary Materials

Electrically-Conductive Polyketone Nanocomposites Based on Reduced Graphene Oxide

Esteban Alejandro Araya-Hermosilla [1,*], Marco Carlotti [1], Francesco Picchioni [2], Virgilio Mattoli [1,*] and Andrea Pucci [3,4,*]

[1] Center for Micro-BioRobotics, Istituto Italiano di Tecnologia Viale Rinaldo Piaggio 34, Pontedera (PI) 56025, Italy; marco.carlotti@iit.it
[2] Department of Chemical Product Engineering, ENTEG, University of Groningen, Nijenborgh 4, 9747AG Groningen, The Netherlands; f.picchioni@rug.nl
[3] Dipartimento di Chimica e Chimica Industriale, Università di Pisa, Via Moruzzi 13, 56124 Pisa, Italy
[4] CISUP, Centro per l'Integrazione della Strumentazione dell'Università di Pisa, Lungarno Pacinotti 43, 56126 Pisa, Italy
* Correspondence: esteban.araya@iit.it (E.A.A.-H.); andrea.pucci@unipi.it (A.P.); virgilio.mattoli@iit.it (V.M.) Tel.: +39 050 883417 (E.A.A.-H.); +39 0502219270 (A.P.); +39 050 883417 (V.M.)

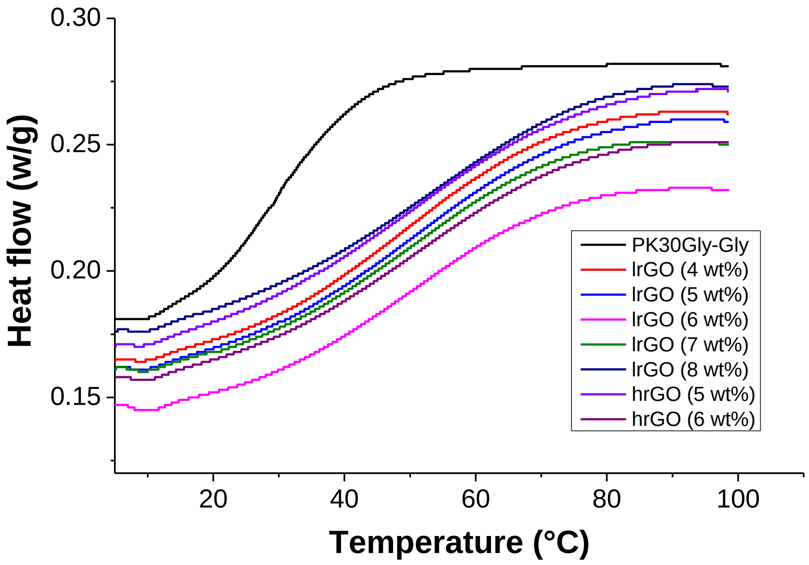

Figure S1. DSC first cycle after thermal history erases of PK30Gly-Gly29 and its respective composite with lrGO and hrGO. The first curves are not shown since they are carried out to remove the thermal history of the polymer and composites.

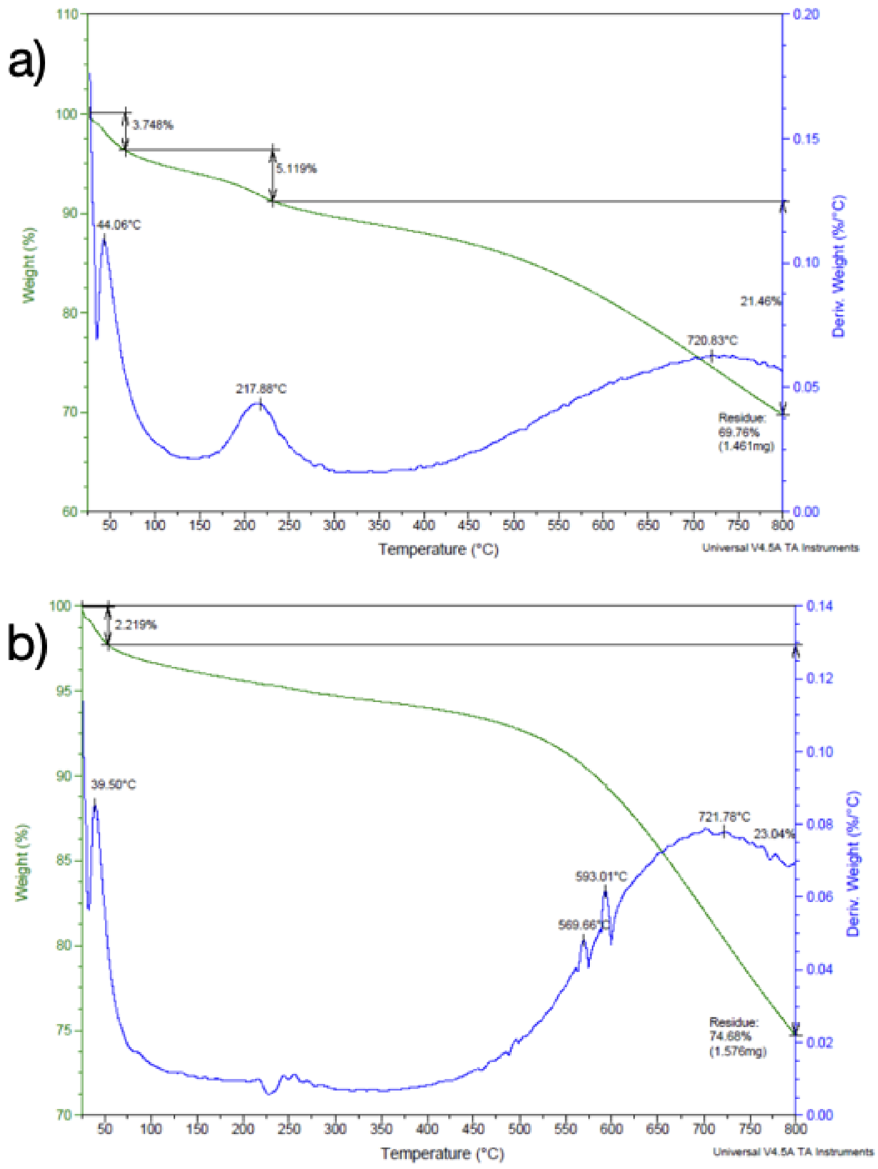

Figure S2. TGA analysis of **(a)** lrGO and **(b)** hrGO.

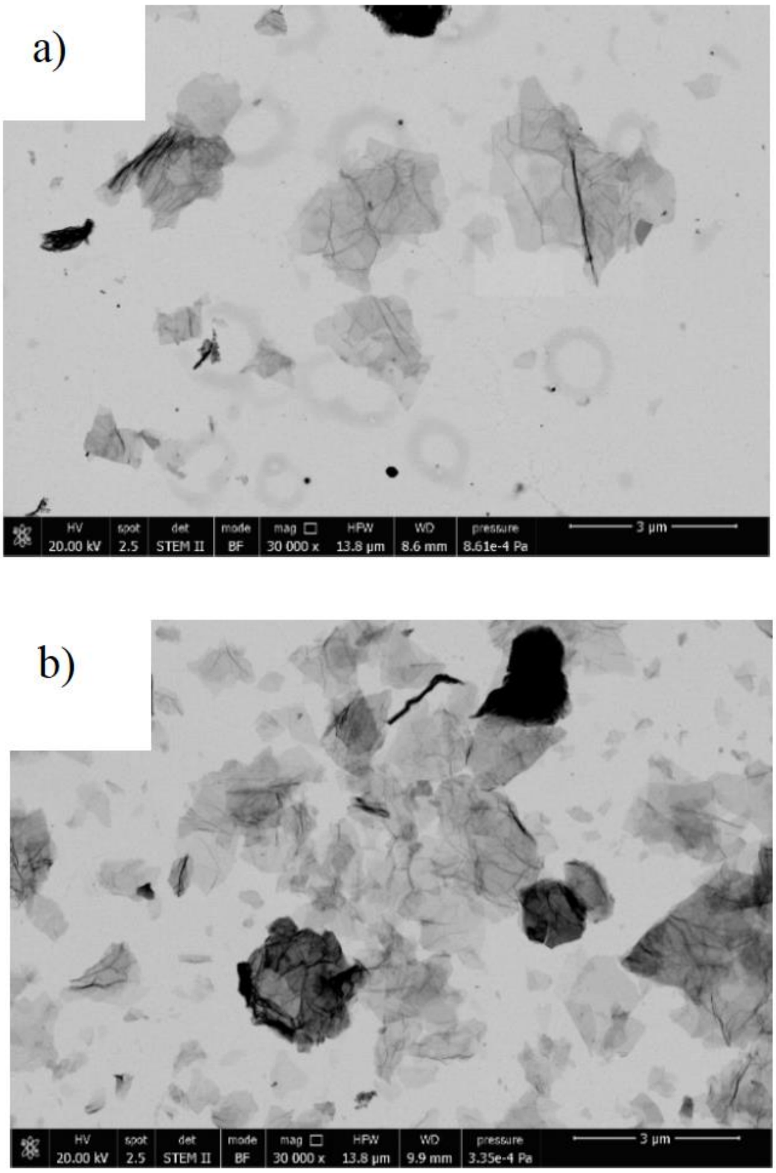

Figure S3. STEM micrograph of (**a**) lrGO and (**b**) hrGO.

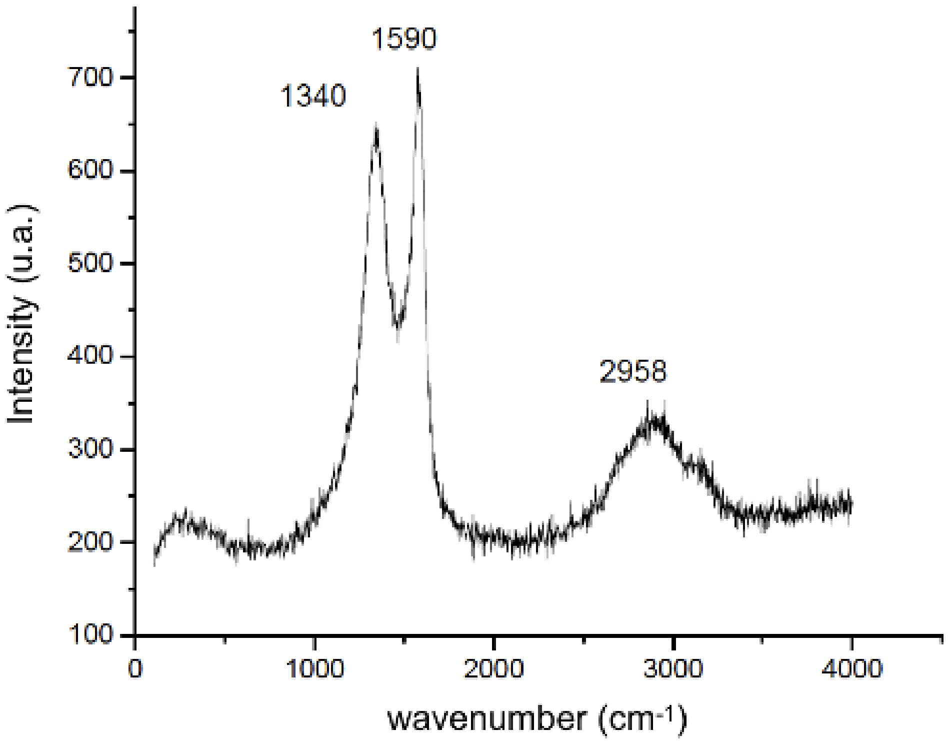

Figure S4. Raman spectrum of lrGO.

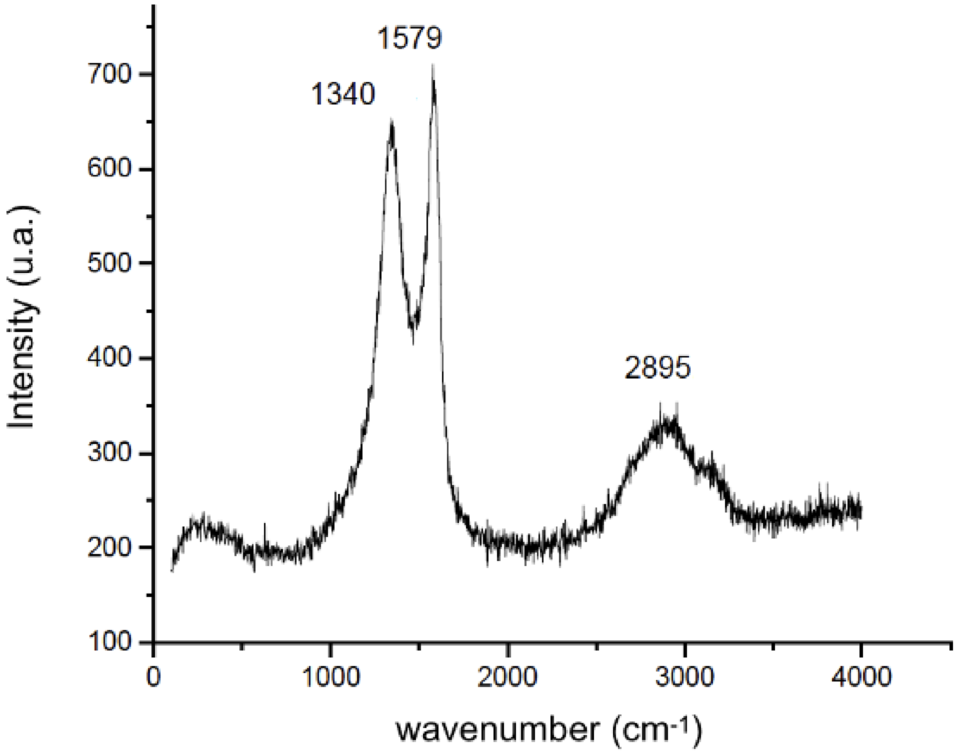

Figure S5. Raman spectrum of hrGO.

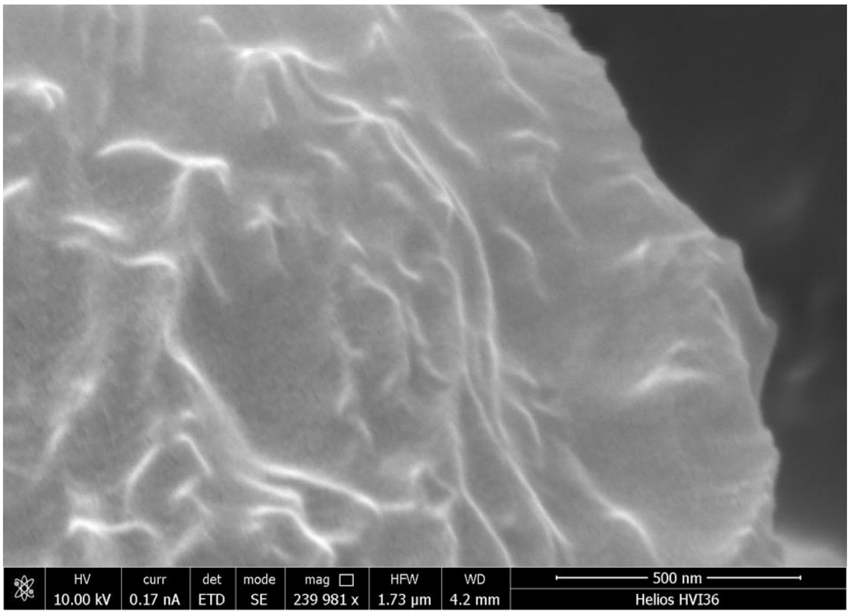

Figure S6. SEM picture of the PK30-Gly-Gly nanocomposite containing 7 wt % lrGO.

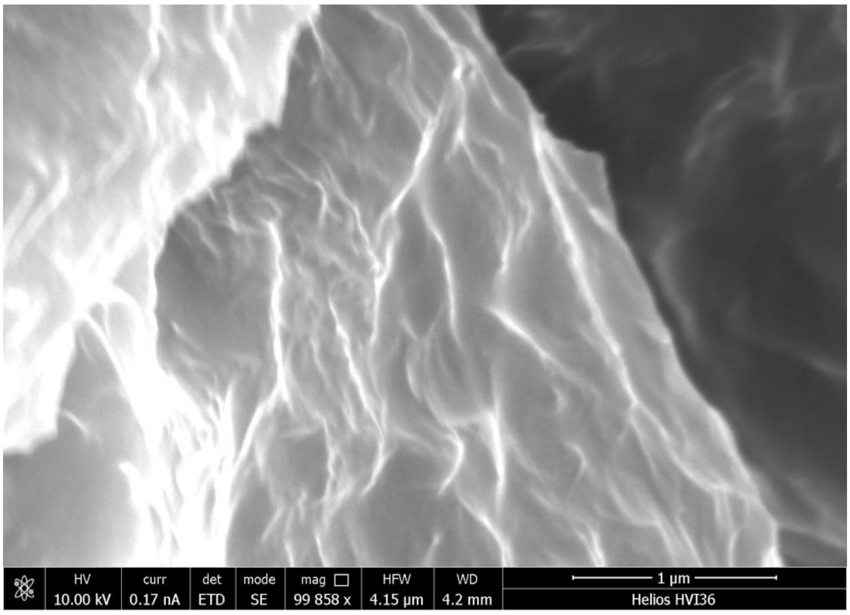

Figure S7. SEM picture of the PK30-Gly-Gly nanocomposite containing 6 wt % of hrGO.

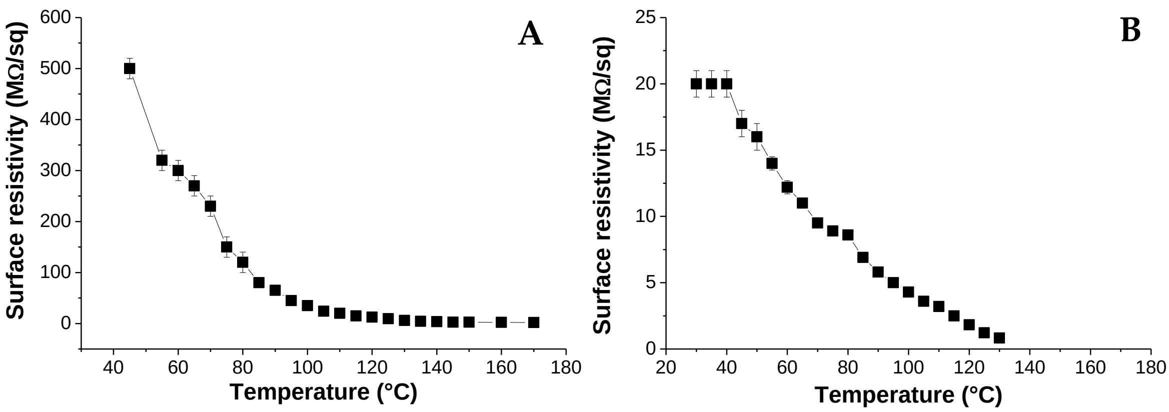

Figure S8. Surface resistivity of the nanocomposite composed by PK30-Gly-Gly and lrGO at different temperatures and filler concentration. (**A**) 4 wt %. (**B**) 5 wt %. Sample thickness of 1.05 mm.
